# Pressure-Driven Dissociation of a Kr Clathrate in the Presence of Colloids

**DOI:** 10.1021/acs.jpclett.6c01558

**Published:** 2026-07-01

**Authors:** Omar A. Arrieta-Guerrero, Angela M. Jaramillo-Granada, José R. Guzmán-Sepulveda, J. C. Ruiz-Suárez

**Affiliations:** Centro de Investigación y de Estudios Avanzados del IPN (Cinvestav), Unidad Monterrey, Vía del Conocimiento 201 PIIT, 66600 Apodaca, Nuevo León, México

## Abstract

Clathrate hydrates (CH) are nonstoichiometric water structures stabilized by hydrophobic molecules, usually at high pressures or low temperatures. Once formed at a certain temperature, their ice-like structures are stable unless the pressure is reduced below critical dissociation values. We explore CH of noble gases using coherence-gated light to perform Fresnel reflectometry (FR) and dynamic light scattering (DLS) in an optically isolated picoliter-sized volume located at the tip of an optical fiber. FR allows us to observe the change in the effective refractive index (*n*_eff_), providing a clear indication of the formation of CH and its porous properties. We found that krypton (Kr) forms CH with higher porosity compared to xenon (Xe), with porosities around 0.54 and 0.02, respectively. Furthermore, by performing DLS measurements, we discovered that in the presence of micrometer-sized particles, Kr CH dissociate with increasing gas pressure. This unexpected phenomenon is due to the Brownian dynamics produced by the Kr atoms accumulated in the highly porous material.

## Graphical abstract

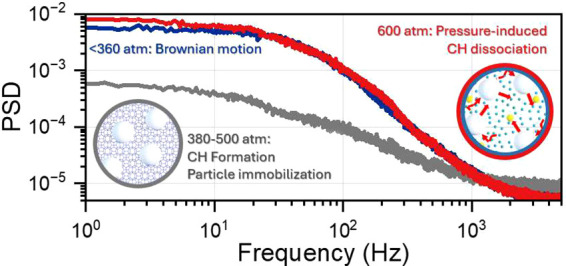

Water solvation is such a strong and persistent phenomenon in nature that even highly hydrophobic molecules succumb to it. The trick used by this universal solvent is to trap such molecules, usually called guests, inside polyhedral water cages, forming clathrate hydrates (CH). The higher the polarizability of the guest molecule, e.g. Xe compared to helium (He), the easier to form stable cages. The phenomenon is quite interesting because symbiosis in physical systems is rather bizarre: A nonpolar guest is suddenly caged by water molecules that, in turn, slow down with the help of the guest, which strengthens the cage. The second law of thermodynamics, at first glance, might appear counterintuitive because both water and guest molecules stop their movement. Despite such entropic penalties, the free energy is reduced. The answer lies in van der Waals forces,^1^ which provide enough cohesion enthalpy between water molecules and guests to compensate for entropy reduction. Two main crystallographic structures of gas CH are distinguished, the von Stackelberg cubic structures CS-I and CS-II, which are formed, respectively, by 8 water cavities (2 small and 6 large) and 24 (16 small and 8 large) occupied by guest molecules^2^ (see Figure 1a).

**Figure 1:**
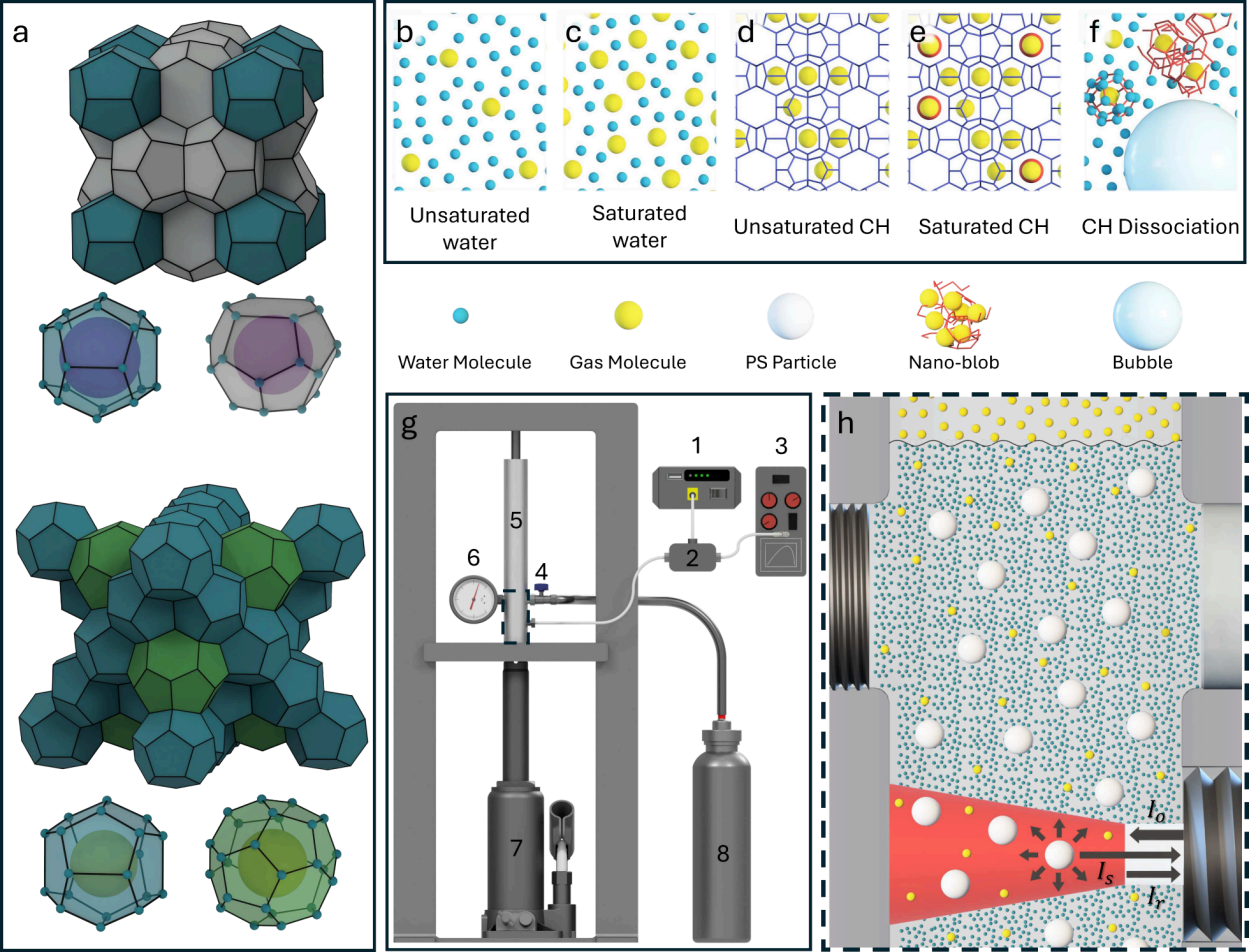
Optical characterization of CH. (a) Unit cell of structures CS-I (top) and CS-II (bottom), formed with Xe and Kr, respectively. (b–f) Schematic representation of pressure-driven CH formation and dissociation. (g) Schematic of the pressurization apparatus instrumented with coherence-gated DLS, where the different components are indicated: broadband light source (1), 1 × 2 multimode splitter used as circulator (2), photoreceiver (3), needle valve (4), stainless steel cylindrical tube where CH formation takes place (5), pressure gauge (6), hydraulic jack (7), and gas cylinder (8). (h) Schematic where light (conical red zone) is carried into the pressurization chamber with an optical fiber, allowing for measurements of the Fresnel reflection at the fiber–medium interface and the dynamic scattering produced by moving particles. For more details, see the text and SI section S1.

CH have two centuries of a rich history of research^3,4^ and, in recent decades, have been at the center of sustainable chemistry studies seeking innovative applications in a wide range of scientific and industrial contexts.^5–7^ For example, significant efforts are being made to reconcile the potential of marine gas CH as an energy resource and their role in climate change.^8,9^ CH are also crucial in specialized applications such as hydrogen storage,^10,11^ carbon dioxide sequestration,^12^ gas separation,^13^ or cryogenic preservation.^14^

Based on a variety of experimental methods and theoretical calculations, we have learned how these structures form.^15–17^ CH are investigated, under different pressure and temperature conditions, by X-ray and neutron diffraction experiments *in situ*;^18–23^ by nuclear magnetic resonance (NMR) and magnetic resonance imaging (MRI), to determine the occupancy of the cage of guest species and their dissociation constants;^24,25^ and by theoretical calculations to understand the physics behind their formation and dissociation.^1,26–28^

The formation and dissociation of CH is, in fact, an important topic investigated by many research groups,^29–33^ where the impact of nanotechnology has recently been explored.^34–36^ Due to their high or low thermal conductivity and specific surface area, a wide variety of nanoparticles have been used to promote^37,38^ or inhibit^39^ CH formation. Whether they are used in one sense or another, the fact is that CH studies with nanoparticles are mostly carried out using thermodynamic experiments. The challenge lies in detecting the very small changes that nanoparticles produce in *T*–*P* curves.^39^

In the present work, instead of using colloidal particles to modify the induction time of CH formation, we used them as tracers to inspect, by means of DLS measurements, the mechanical properties of CH. Although these colloids slightly modify the onset of CH formation, we instead focus on the light scattering they produce. In our experiments, we used three noble gases (He, Kr, and Xe) because they are excellent reference atomic guests. He is taken as a control case (it does not form CH in the conditions of our experiments), whereas Kr and Xe possess properties similar to those of important gases such as methane and carbon dioxide.

First, we used FR to investigate the partition of the gas atoms in the aqueous medium and the onset of CH formation. As pressure increases from 0 to around 500 atm, the effective refractive index *n*_eff_ (or RI), gradually increases from 1.331 (RI of water at 670 nm and 25 °C) to a higher value, depending on the gas used, indicating the formation of a denser solution of water-atoms. Then, a sudden drop in *n*_eff_ suggests the formation of a porous hydrate. In this regard, it should be noted that, once formed and extracted from their containers, porous hydrates have previously been observed through electron microscopy studies.^40,41^ However, in our experiments, they were detected *in situ* when spontaneous formation takes place. Moreover, as the pressure of the system continues to increase, the gas atoms fill the porous CH, as indicated by a growing value of *n*_eff_.

In an independent experiment, we suspended colloidal polystyrene particles (100 nm and 1 μm) before pressurization to perform DLS measurements. Free diffusion is observed at lower pressures, but at the CH transition, the Brownian movement of the tracers ceases completely for the case of Kr, implying their trapping within the CH network. Furthermore, for micrometer-sized colloids, once the porous hydrates are fully filled by gas guests and the pressure is increased further, the CH dissociates, as indicated by the fact that the tracer motion reappears. This intriguing solid–liquid transition is reversible because when pressure is decreased, the CH structure reassembles (immobilizing the colloidal particles again). Finally, if the pressure is decreased to atmospheric conditions, they decay into stable nanostructures entities.^42^

We used a pressure apparatus to dissolve gases in a given volume of water.^42^ It consists of a robust metal structure with a fixed hydraulic jack at the bottom to support and elevate a platform with a cylindrical stainless steel tube, see Figure S1. A 600 atm pressure gauge (Instrutek) and a needle valve (ALCO UN2NS) were attached 5 cm above the bottom on either side of the tube. A stainless steel plunger was attached to the upper part of the structure. To keep high gas pressures, we employed a rubber stopper. The height of the gas column, from the rubber stopper to the water interface, was 28.8 cm. Through the needle valve, the tube is pressurized until a pressure of 10–20 atm is reached. The platform is raised to reduce the volume of the gas and increase the pressure to 600 atm. The experiments were carried out at room temperature (25 °C). See more details in SI section S1.

DLS is an optical technique that allows us to measure the dynamic properties of particulate systems.^43^ When the sample is illuminated, the particles suspended in the medium imprint random fluctuations in the phase of the scattered waves due to their Brownian motion. The autocorrelation characteristics of the resulting stochastic light intensity signal encode the motion of the particles and can be used to retrieve their size distribution, among other dynamical quantities. CG-DLS is a variant of DLS that makes use of spatial and temporal coherence gates to optically isolate a measurement volume of picoliters.^44–46^ From this small volume, singly backscattered light is extracted and amplified in a heterodyne manner with a naturally arising local oscillator. The local oscillator, *E*_r_, emerges from the Fresnel reflection at the fiber–medium interface and *E*_s_ corresponds to light that was singly scattered by the colloidal particles within the coherence volume and coupled back to the optical fiber. In our experimental setup, light from a broadband source (Superlum model BLM-S-670-G-I-4; central wavelength λ_0_ = 670 nm; bandwidth Δλ_0_ = 7 nm) is launched into a 50/50 multimode splitter (Thorlabs model TM50R5F1B, Ø50 μm, 50:50, 0.22 NA) used reversely as a circulator. The multimode optical fibers (MMFs) used are commercially available (Thorlabs M42L05, Ø50 μm, 0.22 NA). The optical signal was measured with a general-purpose photoreceiver (New Focus model 2001-FC) and then digitized with a data acquisition card (National Instruments model NI DAQ 9205). In terms of information retrieval, CG-DLS offers a rich outcome. First, it allows measuring the power spectrum of the light intensity fluctuations, *P*(*f*), which is equivalent to the intensity autocorrelation function measured in DLS.^43^ Also, since *P*(*f*) originates from the contributions of moving scattering elements, its total energy, β = ∫_0_^∞^ *P*(*f*) d*f*, can be used to monitor the strength of dynamic scattering. For monodisperse particles with Brownian motion, β ∝ σ*N*, where σ and *N* are the scattering cross-section and the number density of the scattering elements, respectively. Therefore, CG-DLS allows the following changes in the concentration, size, or optical contrast of the scatterers at multiple scales simultaneously^47,48^ (see the SI). In situations of negligible dynamic scattering, as in the water–gas systems studied here, FR measurements can be translated into a fine refractometric result.^49^ In particular, CG-DLS is self-referenced for these types of approaches since both components of *I*(*t*), time-averaged and fluctuating, are measured simultaneously and continuously. Together, the mechanical plus optical information that can be uniquely accessed in CG-DLS allows us to construct detailed descriptions of multiscale dynamic processes and microrheology in complex fluids.^46,50^ In the present study, we used this rich information to continuously monitor physical changes in gas–water systems as a function of pressure, to clearly identify the onset of CH formation, and for the first time to measure the motion of colloidal particles embedded in the aqueous host as CH form, providing direct insights into their microscopic structural dynamics in a highly pressurized ambient. Further details can be found in SI sections S2–S4, including the calculation of the coherence volume, a numerical verification of the single scattering regime, and the procedure for (optical and mechanical) information retrieval.

Before presenting our findings, it is illustrative to revisit the sequence of events that occur during an experimental cycle of pressurization, CH formation, and depressurization (see Figure 1b–f). As pressure increases, gas atoms are forced to dissolve in the aqueous medium (see Figure 1b,c). Once the gas concentration is high enough, CH nucleation takes place (see Figure 1d,e). When pressure is reduced to atmospheric conditions, CH structures decay into bubbles and stable hydrate nanostructures^42^ (see Figure 1f). We show in Figure 1g a schematic drawing of the apparatus used to carry out the experiments, where the main elements are the pressure cell with a valve to introduce the gas, the mechanism to pressurize it by decreasing the volume, and the optical fiber that enters the cell to guide the entry and exit of light. See full details in SI sections S1 and S2. Finally, in Figure 1h we schematically describe how to perform interferometric measurements of dynamic scattering through an optical fiber, a technique coined as CG-DLS,^46^ which we used in a recent work in high pressure environments.^51^

We studied three noble gases at room temperature: He, Kr, and Xe. It is worth mentioning that instead of employing the usual gas–solid reaction method to create CH (exposing the gas to a finely ground powder of ice),^52,53^ we used a gas–liquid reaction (exposing the gas to a liquid water sample).^42^ In Figure 2a we plot the optical power recovered from Fresnel reflection at the fiber–medium interface. Then, using the equations given in SI section S4, we calculate the *n*_eff_ of the medium (Figure 2b), as a function of pressure, during a cycle of pressurization and depressurization. At the initial pressure (between 10 and 20 atm for the three gases), light (λ = 670 nm) traveling through the fiber core (*n*_g_ = 1.456) is reflected back due to contrast with pure water (*n*_w_ = 1.331).

**Figure 2:**
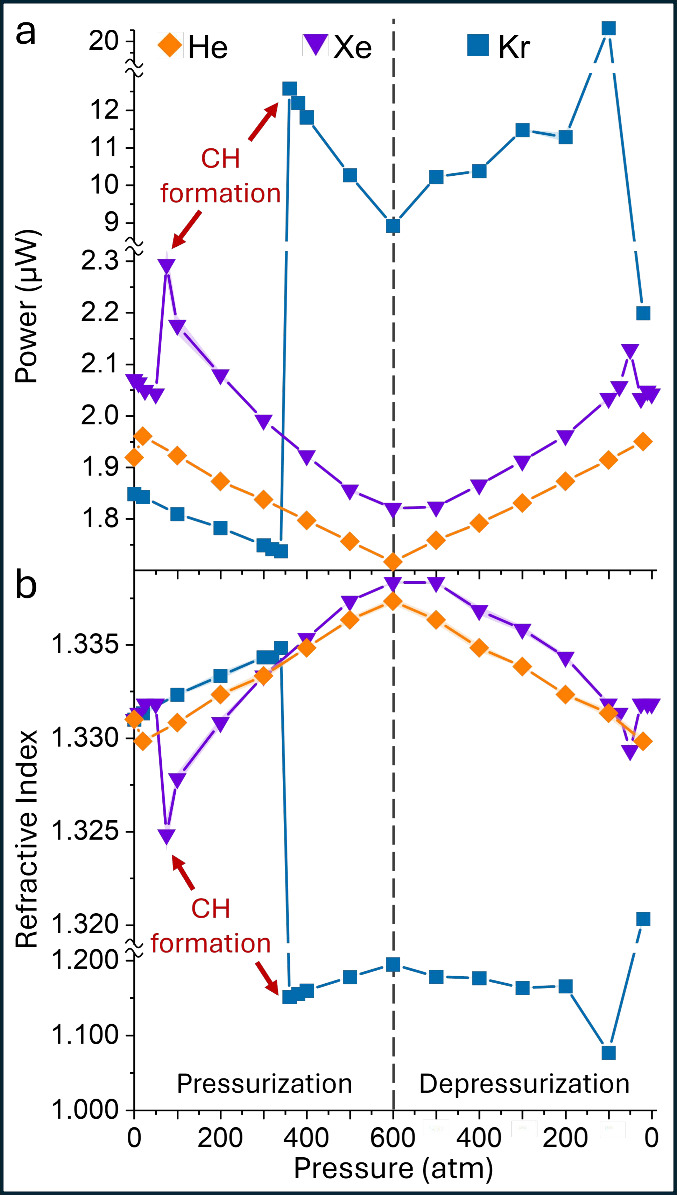
(a) Power of reflected light for the three gases as a function of pressure during a pressurization and depressurization cycle. (b) Refractive index retrieved using Fresnel equations (see SI section S4) as a function of pressure. The sharp drops of the refractive index indicate the formation of CH.

Let us start with He, which we chose as a control because, being a small atom, it does not form CH in the pressure range of our experiments. In fact, at room temperature, even at pressures as high as 1500 atm, He does not form CH.^54^ This water-guest system is expected to behave according to Henry’s law^55^ (which states that the guest concentration in water is proportional to the applied pressure) without exhibiting a phase transition. Consequently, as He is forced to partition into the liquid, the optical power decreases (*n*_eff_ increases). When the system is depressurized, He leaves the aqueous host and the measurement returns to its initial conditions (see Figure 2). The reflected optical power and *n*_eff_ change in this way because water is ”doped” with the additional electronic clouds of the He atoms. In other words, the density of the medium increases with pressure and so does its *n*_eff_. Using 0.38 mM/atm as the Henry constant for He,^56^ the mole fraction of this noble guest in the medium goes from 1.14 × 10^–4^ (at 20 atm) to 3.465 × 10^–3^ (at 600 atm). Thus, assuming that Henry’s law is linear with pressure,^55^ there are 240 water molecules per He atom at the highest pressure. Kr and Xe behave similarly at lower pressures: guest atoms dissolve in the medium as the pressure increases, reporting a medium with higher *n*_eff_. However, at higher pressures, Henry’s law is not linear anymore. At 360 and 75 atm, respectively, an abrupt change occurs: a strong reflection is detected (see Figure 2a), indicating a drop in *n*_eff_ (see Figure 2b). The fact that CH get a lower *n*_eff_ than water is quite interesting, but the very low value for the case of Kr is really intriguing.

It is important to mention that CH, as seen by electronic microscopy, are porous materials.^40,41^ As reported in many other scenarios involving porous materials, emptiness is the reason for low *n*_eff_ which, moreover, can drop significantly with respect to the bulk depending on the degree, scale and geometry of porosity.^57,58^ Large porosities in common materials have been manufactured. For example, silicon dioxide has a refractive index as high as 1.456. However, by using SiO_2_ nanorod arrays, it can be reduced to 1.05,^59–61^ which reflects a decrease of the bulk RI in the first decimal digit (Δ*n* ≈ 0.40). Also with nanorod structures, the effective RI of a layer of indium tin oxide (ITO) can be reduced from 2.06 to 1.34 (Δ*n* ≈ 0.72).^62^ Since the CH formed by Xe and Kr have different structures (Xe forms the structure CS-I while Kr forms CS-II^63,64^ ), the first important conclusion that can be derived from our *n*_eff_ measurements is that the packing of the porous structure CS-II is looser than the packing of the more compact CS-I. The usual way to create CH in the laboratory is to start with finely granulated ice. This is perhaps the reason why CH are porous. However, in our experiments, we did not use granulated ice as a reactor, but rather pure water pressurized with gas. Therefore, the origin of the high porosity must lie elsewhere. We believe that the key lies in the way type CS-II CH stack (see Figure 1a). In other words, stacking unit cells without leaving gaps is practically impossible (unlike when stacking almost perfect cubic cells, such as CS-I type cells).

Interestingly, for the case of Kr, we note that the rate at which *n*_eff_ increases with pressure is significantly different before (1.07 × 10^–5^ ± 3.8 × 10^–7^ atm^–1^) than after (1.8 × 10^–4^ ± 3.4 × 10^–6^ atm^–1^) CH formation. That is, the change of *n*_eff_ per unit change of pressure is almost 17 times larger in solid CH, indicating a more abundant/facilitated entrance of gas atoms in porous hydrate than in liquid water (see SI section S5). Note that the upper and lower scales in Figure 2b are not the same. The high porosity of Kr CH must be the cause of this better partitioning: it is easier to enter a porous solid than a plain liquid or a solid with little porosity, such as Xe CH.

Furthermore, once depressurized, the final value of *n*_eff_ for the Kr case is indicative of the stability of the CH type-II it forms: at atmospheric pressure, *n*_eff_ does not reach the refractive index of bulk water presumably because unit cells remain. Indeed, the CS-II structure has a higher prevalence of planar pentagons that do not strain the hydrogen-bonded structure compared to CS-I. This leads to a lower internal energy, indicating a more energetically favorable (and therefore more stable) structure.^65^

There are different simple models to correlate the *n*_eff_ of an inhomogeneous medium, like the one we are dealing here (solid hydrate + voids), with porosity (see SI section S6). Using the measured *n*_eff_, the porosity values we found are 0.5437 ± 0.0005 and 0.0207 ± 0.0027 for Kr and Xe, respectively. Assuming the coherence volume is estimated to be around 60 pL (60 × 10^15^ Å^3^; see SI section S2) and the volume of the unit cells of type I and type II CH are, respectively, 1700 and 5000 Å^3^,^66^ it contains a bit more than 10^13^ unit cells. Therefore, we can safely assume that what happens in the coherence volume is representative of what happens in the bulk.

The mechanical properties of complex fluids on microscopic scales are commonly measured by means of DLS experiments, in which the mean square displacement (MSD) of suspended colloidal particles is estimated from the autocorrelation characteristics of the intensity fluctuations of scattered light.^43^ The diffusion properties can then be used to estimate the linear viscoelastic moduli.^67,68^ We managed here, for the first time in microrheology science, to perform DLS measurements in highly gas pressurized systems (up to 600 atm). Aqueous suspensions of colloidal particles (polystyrene (PS), 100 nm and 1 μm) were introduced independently into the cell before the experiments. Next, they were subjected to gas pressurization with Kr, in a similar manner as before.

In Figure 3a we plot *P*(*f*), produced by the dynamic scattering from the smallest colloids during a cycle of pressurization and depressurization. As mentioned, *P*(*f*) is equivalent to the intensity autocorrelation function measured in DLS by virtue of the Wiener–Khinchin theorem.^43^

**Figure 3:**
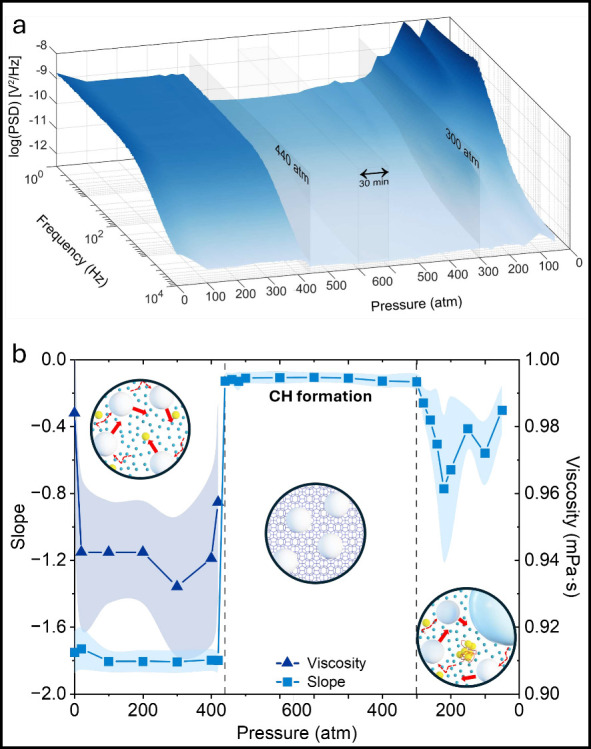
(a) Power spectra vs frequency produced by 100 nm colloidal particles suspended in the medium with dissolved Kr during a pressurization and depressurization cycle. Two measurements taken 30 min apart were recorded at 600 atm. (b) Slopes of the spectra (left) and viscosities (right) of the sample as a function of pressure. The dashed lines indicate the pressure where phase transitions take place. An illustrative representation of the state of the system is presented in each region. The shading in the curves represents the standard deviation of 12 measurements in one run.

The shape of its envelope is a rich source of dynamic information; for instance, a Lorentzian spectrum is indicative of free diffusion in a Newtonian fluid. Moreover, a metric as simple as the asymptotic value of the logarithmic slope (log-slope) encodes the diffusion regime;^69,70^ a value of −2 indicates normal diffusion, while smaller and more negative values can be associated with sub and super diffusion, respectively. From 20 to 420 atm, the logarithmic slope of *P*(*f*) is around −2, indicating that the tracers diffuse freely (see Figure 3b). In this pressure range, retrieving the viscosity of the suspension is valid (see SI section S3) and, as plotted in Figure 3b, is close to that of bulk water. At 440 atm, *P*(*f*) practically vanishes and the log-slope changes to −0.2 (Figure 3b). Tracers are trapped by the CH, so the measured signal is at the level of baseline noise. From 440 to 600 atm, while the porous CH is filled with extra Kr, tracers remain trapped. During depressurization, the guests are released first from the porous CH reservoir and then from water itself. However, like previously observed with the Fresnel intensity, *P*(*f*) does not recover either to its initial condition at 20 atm. Clearly, Kr CH do not dissociate completely, although bubbles start to emerge as evidenced by transients of strong scattering.

Next, we analyze the case of the Kr CH formed in the suspension with 1 μm colloids (Figure 4). Note that in this case, CH formation occurs at 380 atm. We believe that the reason for this lies in the larger area of the colloidal tracers. In fact, compared to smaller colloids, the hydration layers in the micrometer-sized tracers are a hundred times greater, so surface water molecules have less entropy that facilitates the formation of CH. A similar effect has previously been observed in porous materials.^71^ At these lower pressures, the logarithmic slope of *P*(*f*) is also around −2, indicating that the tracers diffuse freely (see Figure 4b). From 380 to 500 atm, *P*(*f*) vanishes as before to the noise floor, making its log-slope change to −0.4, indicating that the tracers are immobilized inside the CH. However, around 600 atm, *P*(*f*) reappears with a logarithmic slope close to −2, revealing the release of the tracers. This outcome is rather unexpected, because according to the dissociation curves of noble gases (see, for example, refs 63 and 72), higher pressure (at the same temperature) implies enhanced stability. This is why, in the absence of colloidal microparticles, the *n*_eff_ shown in Figure 2b does not reflect dissociation.

**Figure 4:**
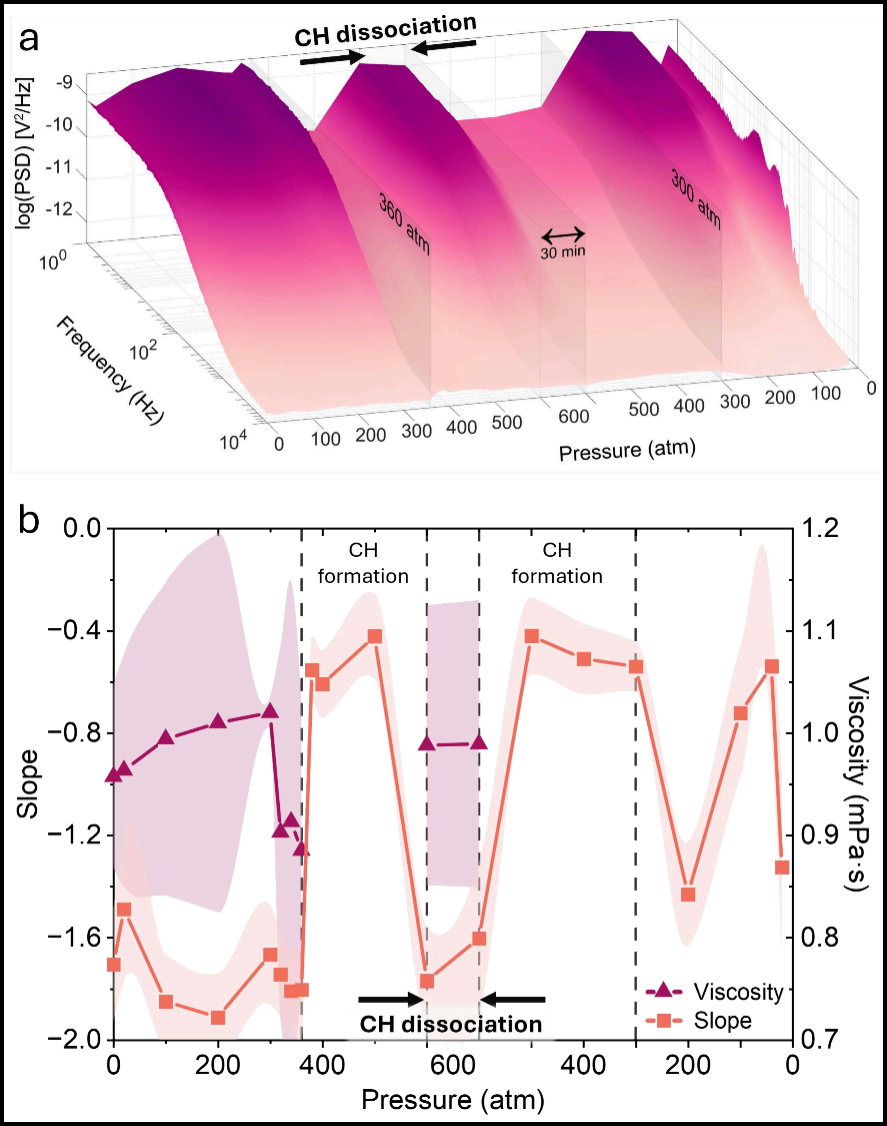
(a) Power spectra vs frequency produced by 1 μm particles suspended in the medium with dissolved Kr during a pressurization and depressurization cycle. Two measurements taken 30 min apart were recorded at 600 atm. (b) Slopes of the spectra (left) and viscosities (right) of the sample as a function of pressure. The dashed lines indicate the pressure where phase transitions take place. The shade in the curves represents the standard deviation of 12 measurements in one run.

A plausible explanation for the dissociation effect we observe is the following: Assuming all 24 cages (16 small and 8 large) are completely filled by Kr, and considering there are 136 water molecules forming the CS-II unit cell, there are 5.66 waters per atom in the unit cell. Since 15 mL of water accounts for a mole fraction of 0.833, the mole fraction of Kr in the medium is then 0.147, which is equivalent to 12.33 g. However, since at the highest pressures (600 atm), the mass of dissolved Kr is close to 30.825 g (considering that the gas above the water column is minimum), where do the extra Kr atoms accommodate? The only possibility is that they enter the pores of the CH, as we mentioned earlier. Interestingly, Kr atoms would be free to move there, as they are not part of the CH structure. Therefore, we claim that such an excess of free Kr atoms, together with the presence of larger colloids, modify the stability conditions. Given that unit cell interactions in CH are weak (they are neither ionic nor covalent, but of dispersive nature), when guest atoms continue to enter the solid, they collide with the embedded colloidal particles. In this scenario, equilibrium is reached immediately since the temperature remains constant. Moreover, the average kinetic energy of the krypton atoms in the pores is $\frac{3}{2}kT$ , which accounts for 3.72 kJ/mol (at 298 K). According to the energy equipartition theorem, this energy is transferred to the colloidal particles. Since this energy is of the same order of magnitude as the cohesion enthalpy of the Kr hydrate (−20 kJ/mol^63^), the hydrate dissociates. It is worth asking two pertinent questions here: (i) how can 3.72 kJ/mol of thermal energy can dissociate a structure whose cohesive energy is −20 kJ/mol? The answer is due to fluctuations, exactly as occurs when one dissolves common salts in water at room temperature even though they have higher cohesive energy than *kT*; (ii) According to the equipartition theorem, the thermal energy of 3.72 kJ/mol is also transferred to the 100 nm colloids, so why do such ten times smaller colloids not dissociate the Kr hydrate as well? We believe that the reason lies in the linear momentum given to the CH lattice. Through a simple calculation, it is easy to show that the average linear momentum of 1 μm colloids is almost 32 times greater than the average linear momentum of 100 nm colloids. A thermodynamic study to observe this dissociation at room temperature is practically impossible to perform because the expected endothermic heat of fusion would be unperceptible to be detected.^39^

As observed in Figure 4b, the phenomenon is reversible. As soon as the cell is depressurized and the molar fraction of Kr decreases (excess Kr vanishes), CH structures form again and remain down to 300 atm. Finally, further depresurization leads to the destabilization of the CH network/lattice and the tracers start diffusing again. However, retrieving the viscosity in this pressure range is not possible since the measured power spectra is distorted due to the buoyant bubble dynamics (Figure 4b).

We carried out similar experiments for Xe CH (data not shown). The light scattering signal produced by both small and large tracer particles persisted at all pressures, suggesting that Xe CH, which form at 75 atm in pure water, do not form in suspensions. In other words, they are unstable because of the colloidal presence. The different impact of the particles on the CH nucleation conditions may be explained by their specific structure. CH Xe has a less energetically favorable arrangement with respect to water molecules at the vertices of the cages compared to CH Kr,^65^ which would suggest a structure more susceptible to perturbations caused by colloidal agitation. When the cell is depressurized back to atmospheric pressure and equilibrium is reached (bubbling dynamics ends), the different CH systems decay into a suspension of hydrate nanostructures, which are stable in the long term, and have been previously reported by us in a previous article.^42^

Noble gas CH were optically studied by performing measurements of FR and DLS as a function of pressure and at room temperature. The onset of CH formation can be clearly identified in both measurements. From FR, we were able to determine the difference in porosity related to the type of CH structure formed by Kr and Xe. He was used as a control guest because it does not form stable CH in the pressure range of our experiments; it only produces a smooth increase in the RI of the aqueous medium as a result of the inclusion of gas atoms. Furthermore, in the case of Kr, CH were also formed in the presence of colloidal tracers to access their microrheological properties on the basis of measurements of the scattered light. The possibility of performing, for the first time, DLS measurements at such highly pressurized gas systems offers great experimental advantages not only for CH research but also for studying dynamic systems in highly pressurized environments. The most intriguing finding in our study is the dissociation of the Kr CH at a pressure higher than its dissociation value in the presence of micrometer-sized colloids. This effect is reversible and, although it does not seem to point toward new types of interactions, it offers a unique mechanism for controlling the assembly and dissociation of CH. Finally, in future experiments, we would like to explore other gases to determine whether the observed dissociation of CH in pressurized colloidal suspensions also occurs at lower temperatures.

Although the results of our research constitute significant discoveries in themselves, the unexpected outcome of dissociation could be investigated further to offer some future insights, for example, in the fabrication of viscous CH materials. Perhaps, by adding colloidal particles to the medium, we could modify their mechanical properties rapidly by increasing the pressure. In cryogenic applications for cell preservation, we could freeze or thaw the storage medium by pressure modulation. The fusion transition that we discovered is rapid compared to the dissociation times with increasing temperature or decreasing pressure. Our group’s future work will consist of studying the influence of colloidal particles in CH of gases such as methane and carbon dioxide, which are relevant in energy applications.

## Supplementary Material



## Notes

Section S1 shows photographs of the device for the optical characterization of noble gas clathrate hydrates; Sections S2–S5 discuss the experimental details for data acquisition and the calculation of physical parameters; Section S6 discusses effective medium models to calculate the porosities of clathrate hydrates (PDF)
