# Supplementary material for: Pressure-Driven Dissociation of a Kr Clathrate in the Presence of Colloids
Source: J Phys Chem Lett. 2026 Jul 1;17(28):7879–86. doi: 10.1021/acs.jpclett.6c01558 (PMC13383828; doi:10.1021/acs.jpclett.6c01558)
Supplement: Supplementary file 1 [file jz6c01558_si_001.pdf]

Supporting Information

Pressure-driven Dissociation of a Kr Clathrate in the Presence of Colloids

Omar A. Arrieta-Guerrero, Angela M. Jaramillo-Granada, José R. Guzmán-Sepúlveda*, and J. C. Ruiz-Suárez#

CINVESTAV-Monterrey, PIIT, Apodaca, Nuevo León, 66628, México.

*jose.guzmans@cinvestav.mx; #cruiz@cinvestav.mx

S1. Experimental setup

Fig. S1 shows photographs of our experimental setup for the optical characterization of noble gas clathrate hydrates. Essentially, it consists of a high-pressure chamber, previously used to study the formation of nanostructured water-gas domains when gases dissolve at high pressures [1], adapted to be instrumented with the CG-DLS technique [2]. The pressurization apparatus consists of a metallic frame attached to a hydraulic jack at the bottom used to raise a platform onto which a stainless-steel chamber is mounted (Fig. S1(a)). The chamber has a cylindrical shape, 38cm-long, with external and internal diameters of 3.81 cm and 1.27 cm, respectively. The plunger at the top seals the chamber with a rubber stopper and is inserted into the chamber as the platform is raised (Fig. S1(a)). A needle valve (ALCO UN2NS) and a 600 atm pressure gauge (Instrutek) were installed 5 cm above the inner bottom of the chamber (Fig. S1(b)). In the third port, a customized seal screw is placed with the endoscopic-like optical fiber probe of CG-DLS (Fig. S1(b)). A stainless-steel screw was perforated along its length to allow an optical fiber to pass through it; then the fiber was fixed with high-pressure epoxy (Fig. S1(b)). This optical fiber is part of the CG-DLS setup, and it is used to carry the light from the broadband light source to the sample and to collect light from the sample for its subsequent detection (New Focus photoreceiver model 2001-FC).

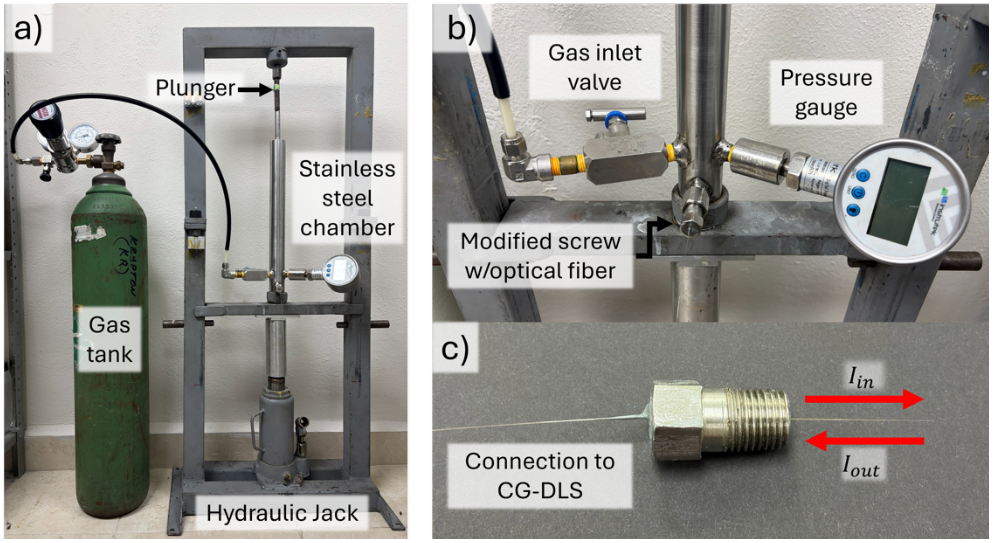

Figure S1. Experimental setup for the optical characterization of noble gas clathrate hydrates with CG-DLS. (a) Photograph of the pressurization system. (b) Close-up of the ports of the pressure chamber for gas input, pressure gauge, and the endoscopic-like optical fiber probe of CG-DLS. (c) Photograph of the modified seal screw with the optical fiber mounted to monitor the inside of the pressurized chamber.

S2. Size of the coherence volume.

In our experimental setup, a commercial broadband light source (Superlum model BLM-S-670-G-I-4) was used with central free-space wavelength $\lambda_0 = 670\ \mathrm{nm}$ and spectral bandwidth $\Delta\lambda = 7\ \mathrm{nm}$. According to the manufacturer's specifications, its emission spectrum has approximately a Gaussian envelope; therefore, its coherence length, l_c, can be estimated as $l_c = \sqrt{2\ln(2)/\pi}\ (\lambda_0^2/(n\Delta\lambda))$ [3], which results in $l_c \approx 30\ \mu\mathrm{m}$ for aqueous media ($n \approx 1.33$). The light from the source was launched into a 50/50 multimode splitter (Thorlabs model TM50R5F1B, Ø50 µm, 50:50, 0.22 NA) used in reverse as a circulator. The multimode optical fibers (MMFs) used are commercially available (Thorlabs M42L05, Ø50 µm, 0.22 NA). Considering the size of the core of the MMFs used ($50\ \mu\mathrm{m}$), the coherence volume from where the scattering signal is extracted, is on the order of $V_c \approx 60\ \mathrm{pL}$.

S3. Information retrieval.

In CG-DLS, the light from the broadband source travels along the core of the optical fibers until it reaches the sample. At the fiber-medium interface, most of the incident field, E_0, is transmitted into the sample, E_t, while the rest is reflected to the fiber's core, E_r. In the presence of scattering elements within the coherence volume, a portion of E_t is scattered and coupled back to the core of the optical fiber, E_s. In general, the intensity detected is a time-fluctuating signal that results from the interference of E_r and E_s, which travel together to the detector, namely $I(t) = |E_r + E_s|^2 = I_r + I_s + 2\sqrt{I_r I_s}\cos(\Delta\phi(t))$, where $I_{r,s}$ is the time-averaged intensity of $E_{r,s}$, respectively; $\Delta\phi(t)$ is the random phase difference between the two interfering fields, whose temporal variations are determined by the moving particles. In this way, the practical implementation of CG-DLS can be described as an optical fiber-based, common-path interferometer operating in reflection, where the coherence volume is localized at the tip of the optical fiber probe.

In most practical situations, $|E_r| \gg |E_s|$; thus, the intensity detected can be expressed as $I(t) \approx I_r + 2\sqrt{I_r I_s}\cos(\Delta\phi(t))$. With this simplification, it is evident that $I(t)$ consists of a static component (first term) and a dynamic one (second term), and that E_r acts both as a baseline, reference field and as a local oscillator for the interferometric amplification of E_s.

The main use of CG-DLS has relied on the second term since it contains dynamic information equivalent to that measured in DLS [4]. The envelope of the power spectrum of the light intensity fluctuations, $P(f)$, encodes the particles' dynamics; it can be used to retrieve either their size distribution or, if the particles are known, their diffusion properties and the viscoelasticity of the suspending medium [5, 6]. These aspects of the measurement have been widely explored in various scenarios both in aqueous media [2] and, more recently, in aerosols [7].

Here, we used this piece of information to estimate the viscosity of the gas-water medium as a function of pressure, as follows. Each spectrum was decomposed into a collection of discrete Lorentzian components, $P(f) = (2/\pi)\sum_{i=1}^{N}(a_i\,\nu_i)/(f^2 + \nu_i^2)$, where f is the frequency; ν_i and a_i is the corner frequency and the relative amplitude of each Lorentzian function, respectively, with $\sum_{i=1}^{N} a_i = 1$. Two Lorentzian components ($N = 2$) were sufficient to describe the envelope of $P(f)$ in all cases; one of them carried most of the weight while the second one adjusted only a small portion of $P(f)$ close to the noise floor. ν_i is the inverse of τ, the characteristic autocorrelation time measured in DLS [4]. With it, the diffusion coefficient of the colloidal particles can be estimated as $D_{eff} = 2\pi/(q^2\tau)$, where q is the magnitude of the scattering vector $q = 4\pi n\sin(\theta/2)/\lambda_0$, with n being the refractive index of the suspending medium, θ the scattering angle ($n \approx 1.331$ and $\theta =$

π rad in our case), and λ_0 is the free-space wavelength of the incident light. Since the probe particles are known, D_{eff} can then be used to estimate the effective viscosity of the suspending medium, $\eta = k_B T / 3\pi d D_{eff}$, where $k_B T$ is the thermal energy, with k_B being Boltzmann's constant and T the absolute temperature, and d is the diameter of the particles.

S4. Fresnel reflectometry with CG-DLS.

A unique, less-explored aspect of CG-DLS is its potential for retrieving optical information. In situations where E_s is negligible or absent, the time-averaged intensity, $\langle I(t) \rangle_t$, is determined by E_r which in turn depends directly on the optical contrast of the fiber-medium interface, $\langle I(t) \rangle_t \approx I_r = |E_r|^2$. This piece of information is not available in traditional DLS setups due to their homodyne operation, where E_s is measured directly. More importantly, when E_s is negligible, a reflectometric measurement can be translated into a refractometric outcome since E_r emerges from the Fresnel reflection at the fiber-medium interface. In other words, because the refractive index (RI) of the optical fiber is known, this intensity measurement allows estimating the effective RI of the sample.

The signal processing is straightforward, first, because the voltage signal delivered by the photo-receiver (New Focus model 2001-FC) is proportional to the incident optical power which, in turn, is proportional to the Fresnel reflectivity, R i.e., $P \propto I_r \propto R$. For the situation at hand, where dispersion effects can be neglected over the bandwidth of the light source and light propagation takes place close to the optical axis, the Fresnel reflection coefficient at the fiber-medium interface, R, can be approximated to the case of a plane wave at normal incidence:

$$R = \left| \frac{n_1 - n_2}{n_1 + n_2} \right|^2$$

where $n_{1,2}$ is the RI of each material, fiber and medium, respectively. In the initial conditions of our experiments, where the fiber is immersed in DI water, the baseline reflectivity is:

$$R^{(0)} = \left| \frac{n_{fiber} - n_{water}}{n_{fiber} + n_{water}} \right|^2$$

whose value is around $R^{(0)} \approx 0.21\%$, considering that, at $\lambda_0 = 670\ \mathrm{nm}$ and $\mathrm{T} = 25°\mathrm{C}$, the RI of water and silica is $n_{water} \approx 1.331$ [8, 9] and $n_{fiber} \approx 1.456$ [10, 11].

Changes in the RI of water can be accounted as small perturbations to its baseline value as:

$$R_{eff} = \left| \frac{n_{fiber} - (n_{water} + \delta n)}{n_{fiber} + (n_{water} + \delta n)} \right|^2 = \left| \frac{n_{fiber} - n_{eff}}{n_{fiber} + n_{eff}} \right|^2$$

where δn represents the change in RI, while $n_{eff} = n_{water} + \delta n$ denotes the effective RI of water in the new conditions, and R_{eff} its corresponding reflectivity. By taking the baseline condition as reference, and knowing that the optical power detected is proportional to the Fresnel reflectivity, n_{eff} can be found numerically, using the ratio of the power measured at the two conditions, $P^{(0)}$ and P_{eff}, as follows:

$$\left| \frac{n_{fiber} - n_{eff}}{n_{fiber} + n_{eff}} \right|^2 = \frac{\left| \frac{n_{fiber} - n_{water}}{n_{fiber} + n_{water}} \right|^2}{\left(\frac{P^{(0)}}{P_{eff}} \right)}$$

In this equation, the only unknown is n_{eff}. Once again, n_{fiber} and n_{water} correspond to the baseline condition and their values are taken from the literature, while the optical powers are measurable quantities in our experiments. In practice, once the optical power is measured at the two conditions, n_{eff} is numerically swept until the difference between both sides of the equation is minimized.

In the experiments, at each pressure, we let the system stabilize for 5 minutes before acquiring data; then, data were recorded for 60 seconds, with sampling frequency of 200 kHz and integration time of 5 seconds, which results in twelve independent realizations per pressure point. The mean and standard deviation of such realizations is plotted in Fig. 2(a) (data points and shaded region around them, respectively). As can be seen, variability is minimal; for instance, at the onset of hydrate formation, the mean optical power and standard deviation for Kr and Xe after stabilization was $12.5788 \pm 0.0025\ \mu\mathrm{W}\ (\mathrm{CV} = 0.0199\%)$ and $2.2937 \pm 0.0291\ \mu\mathrm{W}(CV = 1.2687\%)$, respectively.

n_{eff} was calculated using the mean value of optical power. To propagate the variability of the optical power to the RI, lower/upper boundaries were established by retrieving n_{eff} using the mean optical power +/- one standard deviation. The mean and boundary values of RI correspond to the markers and shaded region around data points in Fig. 2(b), respectively. Based on these estimations, at the pressure of hydrate formation, the RI for Kr and Xe is 1.1512 ± 0.0002 and 1.3248 ± 0.0009, respectively.

This refractometric capability of CG-DLS has been exploited to measure the polarizability of proteins in aqueous solution, being able to detect fine RI variations down to the fourth decimal digit [12]. In the present study, we used the rich outcome of CG-DLS to continuously monitor physical changes in gas-water systems as a function of pressure, to clearly identify the onset of clathrate formation and, for the first time, to measure the motion of colloidal particles embedded in the aqueous host as clathrates form, providing direct insights of their microscopic structural dynamics.

S5. Entrance rates of the noble gases

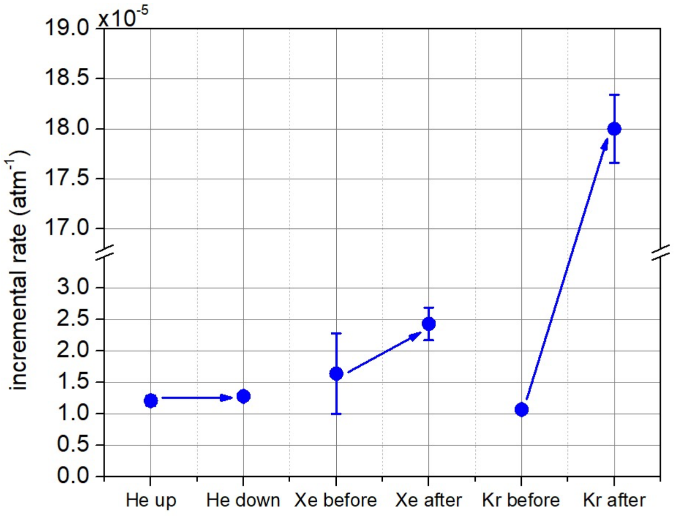

Figure S2. Entrance rates of He to water, and Xe and Kr to water (before the CH transition), and to the CH solids.

S6. Effective medium models

After estimating n_{eff} from our measurements, we used an effective medium approach to calculate the porosity, ϕ, around the onset of CH formation. We considered CH as porous materials consisting of vacuum inclusions embedded in a continuous matrix, which in turn is composed by the water–gas mixture present at the condition of CH nucleation. In our situation, all constituents are dielectric; therefore, the RI and the relative permittivity, ε, are related as $\varepsilon = n^2$. The calculations were done by considering the values of RI retrieved from the measurements before and after CH nucleation (data points in Fig. 2(b) marked with roman numbers II and III, respectively).

We used several effective medium models (EMM) that have been previously reported to describe the optical properties of nanoporous materials, including the well-known Maxwell-Garnett theory (MG) and the asymmetrical Bruggeman model (AB) [13, 14] as well as more refined formulations like the volume averaging theory (VAT) [15, 16], the Looyenga model (LM) [17], and del Rio-Zimmerman-Dawe model (dRZD) [18]. Our reasoning to consider these models is the following.

MGT was originally formulated for monodispersed spherical inclusions, of size much smaller than the wavelength, and arranged in a cubic lattice structure; it is valid for porosities $\phi \leq 52\%$. This is the most popular model, and we included it for reference i.e., $\left(\frac{\varepsilon_{eff}-\varepsilon_c}{\varepsilon_{eff}+2\varepsilon_c}\right) = \phi\left(\frac{\varepsilon_d-\varepsilon_c}{\varepsilon_d+2\varepsilon_c}\right)$. ABM considers polydisperse spherical inclusions and it is applicable to the full range of porosity, $0 \leq \phi \leq 1$ i.e., $1-\phi = \left(\frac{\varepsilon_{eff}}{\varepsilon_c} - \frac{\varepsilon_d}{\varepsilon_c}\right) / \left[\left(\frac{\varepsilon_{eff}}{\varepsilon_c}\right)^{1/3}\left(1-\frac{\varepsilon_d}{\varepsilon_c}\right)\right]$. VAT was derived by volume averaging Maxwell's equations and disregards the inclusions shape, size, and spatial distribution; for dielectric materials, it takes the simple form of a volume fraction-weighted average for ε i.e., $\varepsilon_{eff} = (1-\phi)\varepsilon_c + \phi\varepsilon_d$, and it has been shown to describe rigorous numerical simulations well [13]. LM, $\varepsilon_{eff}^{1/3} = (1-\phi)\varepsilon_c^{1/3} + \phi\varepsilon_d^{1/3}$, and dRZD, $n_{eff} = n_c\left[1+\phi\left(\sqrt{n_d/n_c}-1\right)\right]/\left[1+\phi\left(\sqrt{n_c/n_d}-1\right)\right]$, consider no particular shape for the inclusions and are best suited for materials with high porosities, arbitrary microstructure, and high RI contrast between the inclusions and the matrix e.g., porous silicon [19]. In the equation for each model, the subscripts "c", "d", and "eff" indicate, respectively, the continuous phase (matrix), the dispersed phase (pores; $\varepsilon_d = n_d^2 = 1$ in all cases), and the effective property.

The mean and limiting values of porosity were determined by calculating ϕ using the mean and boundary values of n_{eff} in the different EMM (see details in Section S4). Table S1 summarizes the estimations of the mean porosity. All EMM yield similar results; directly averaging those mean values results in average porosity of $\phi \approx 0.5437 \pm 0.0258$ for Kr (EMM variability within 5%) and $\phi \approx 0.0208 \pm 0.0021$ for Xe (EMM variability within 10%).

The boundary values of n_{eff} produce different internal variability metrics within each EMM (not reported) since each EMM accounts for n_{eff} in a different way e.g., as RI, permittivity, or permittivity elevated to some power. Therefore, a more rigorous measure of global variability can be established from the average variance across all models, as $\sigma = \sqrt{\sum_i \sigma_i^2/N}$, where σ_i is the variability reported by the i-th model and $N = 5$ models. Overall, this results in porosity values of 0.5437 ± 0.0005 and 0.0207 ± 0.0027 for Kr and Xe, respectively.

Table S1. Porosity estimation with different effective medium models (MG: Maxwell-Garnett; AB: asymmetrical Bruggeman; VAT: volume averaging theory; LM: Looyenga model; dRZD: del Rio-Zimmerman-Dawe model). See text for details.

	CH pressure formation (atm)	Before CH nucleation		After CH nucleation		Porosity, ϕ				
		n_c	ε_c	n_{eff}	ε_{eff}	MG	AB	VAT	LM	dRZD
Kr	360	1.3348	1.7817	1.1512	1.3253	0.5451	0.5408	0.5839	0.5364	0.5124
Xe	75	1.3318	1.7737	1.3248	1.7551	0.0206	0.0206	0.0240	0.0202	0.0183

References

[1] A. M. Jaramillo-Granada, A. Reyes-Figueroa, and J. Ruiz-Suárez, "Xenon and krypton dissolved in water form nanoblobs: No evidence for nanobubbles," *Physical review letters,* vol. 129, no. 9, p. 094501, 2022.

[2] J. Guzman-Sepulveda and A. Dogariu, "Probing complex dynamics with spatiotemporal coherence-gated DLS," *Applied optics,* vol. 58, no. 13, pp. D76-D90, 2019.

[3] C. Akcay, P. Parrein, and J. P. Rolland, "Estimation of longitudinal resolution in optical coherence imaging," *Applied optics,* vol. 41, no. 25, pp. 5256-5262, 2002.

[4] B. J. Berne and R. Pecora, *Dynamic light scattering: with applications to chemistry, biology, and physics*. Courier Corporation, 2000.

[5] T. Mason, H. Gang, and D. Weitz, "Rheology of complex fluids measured by dynamic light scattering," *Journal of Molecular Structure,* vol. 383, no. 1-3, pp. 81-90, 1996.

[6] T. G. Mason, "Estimating the viscoelastic moduli of complex fluids using the generalized Stokes–Einstein equation," *Rheologica acta,* vol. 39, pp. 371-378, 2000.

[7] E. Zora-Guzman and J. R. Guzman-Sepulveda, "Optical characterization of native aerosols from e-cigarettes in localized volumes," *Biomedical Optics Express,* vol. 15, no. 3, pp. 1697-1708, 2024.

[8] M. Daimon and A. Masumura, "Measurement of the refractive index of distilled water from the near-infrared region to the ultraviolet region," *Applied optics,* vol. 46, no. 18, pp. 3811-3820, 2007.

[9] G. M. Hale and M. R. Querry, "Optical constants of water in the 200-nm to 200-μm wavelength region," *Applied optics,* vol. 12, no. 3, pp. 555-563, 1973.

[10] I. H. Malitson, "Interspecimen comparison of the refractive index of fused silica," *Josa,* vol. 55, no. 10, pp. 1205-1209, 1965.

[11] Y. Arosa and R. de la Fuente, "Refractive index spectroscopy and material dispersion in fused silica glass," *Optics Letters,* vol. 45, no. 15, pp. 4268-4271, 2020.

[12] J. R. Guzman-Sepulveda, R. Wu, A. P. Kalra, M. Aminpour, J. A. Tuszynski, and A. Dogariu, "Tubulin polarizability in aqueous suspensions," *ACS omega,* vol. 4, no. 5, pp. 9144-9149, 2019.

[13] M. M. Braun and L. Pilon, "Effective optical properties of non-absorbing nanoporous thin films," *Thin Solid Films,* vol. 496, no. 2, pp. 505-514, 2006.

[14] T. Galy, M. Marszewski, S. King, Y. Yan, S. H. Tolbert, and L. Pilon, "Comparing methods for measuring thickness, refractive index, and porosity of mesoporous thin films," *Microporous and Mesoporous Materials,* vol. 291, p. 109677, 2020.

[15] J. De Rio and S. Whitaker, "Maxwell's equations in two-phase systems I: Local electrodynamic equilibrium," *Transport in Porous Media,* vol. 39, no. 2, pp. 159-186, 2000.

[16] J. d. Río and S. Whitaker, "Maxwell's equations in two-phase systems II: Two-equation model," *Transport in Porous Media,* vol. 39, no. 3, pp. 259-287, 2000.

[17] H. Looyenga, "Dielectric constants of heterogeneous mixtures," *Physica,* vol. 31, no. 3, pp. 401-406, 1965.

[18] J. Del Rio, R. Zimmerman, and R. Dawe, "Formula for the conductivity of a two-component material based on the reciprocity theorem," *Solid state communications,* vol. 106, no. 4, pp. 183-186, 1998.

[19] D. Estrada-Wiese and J. A. del Río, "Refractive index evaluation of porous silicon using bragg reflectors," *Revista mexicana de física,* vol. 64, no. 1, pp. 72-81, 2018.
